# Impact of Combined modular assessment on deep learning and personal development of medical students

**DOI:** 10.12669/pjms.321.9007

**Published:** 2016

**Authors:** Shabana Ali, Humaira Fayyaz Khan

**Affiliations:** 1 Dr. Shabana Ali, Assistant Professor of Anatomy, Islamic International Medical College, Riphah International University, Islamabad, Pakistan; 2 Dr. Humaira Fayyaz Khan, Associate Professor of Physiology, Islamic International Medical College, Riphah International University, Islamabad, Pakistan

**Keywords:** Assessment, Performance, Medical students

## Abstract

**Objective:**

To determine the effect of transition of summative assessment from frequent modular to combined block assessment and its role on learning of medical students.

**Methods:**

A descriptive study was carried out at Islamic International Medical College. A questionnaire comprizing of 8 questions with Likert scale (1-5) was designed for 3^rd^ year students. The questions were grouped in three categories which included learning acquired, personal development and efficacy of assessment tools used in combined block assessment and frequent modular exam separately. Results of end of year exams were analyzed for difference in performance of students in two exams. The received data was analyzed by using SPPS 21.

**Results:**

About 60% students agreed that they need to study regularly in frequent modular exams. Combined block assessment promoted more indepth studies and multiple revisions 51% and 55% students respectively. About 42% students, in comparison with 33%, agreed that CBA helped in their personal development while 42% students agreed to assessment tools used in CBA while only 28% students to those used in frequent modular exam. About 47% students agreed that assessment tools in CBA were useful for deep learning and 47% students agreed that time given in CBA was enough in exam preparation. Comparison of all results (x 2 tests) was statistically significant. The comparison of end of year performance showed improvement in the mean of total marks obtained and decrease in the number of failed students in combined block assessment.

**Conclusion:**

Transition from frequent to combined block assessment with regular formative assessment has positive effect on learning, personal grooming and performance in summative assessment.

## INTRODUCTION

Summative assessment is aprocess to assess the learning outcomes.^1^ Assessment drives learning and is seen as an important part of educational process. It not only outlines the quality of the students but it also steer’s the learning and behavior of students and helps them to learn effectively.^2^ Summative assessment is outcome based and is planned to give students specialized self-regulation and liability while formative assessment is for learning and it underpins intrinsic drive in students to learn and inspire them to set higher standards for themselves.^3^

It is imperative to understand that students adopt different learning strategies according to their own needs.^4^ A student may be a surface or deep learner. Deep learners integrate newly acquired and preexisting knowledge in a better way which gives them better understanding of the subject. It is encoded and decoded with great depth and is associated with higher educational accomplishments. The surface learners, on the other hand, intend to learn only those portions of subject which they think might come in the exams. These students are inclined to put emphasis on certain specific and important facts of subject which may not be linked concept.^5^ This type of learning may be associated with extra undue workload with little independence in learning while deep learning can be linked with good quality teaching with effective feedback and freedom in learning^6^ and clear awareness of learning objectives. An inappropriate assessment method may enforce an undue stress on a student to take wrong approach towards learning. Regular formative assessment with constructive feedback may help these students. Nevertheless, assessment should always be fair, reliable and properly conducted.^7^

Integrated modular system usually revolves around frequent assessments both formative and summative. They consume considerable resources and give little preparation time to the students while faculty is busy most of the time in designing question papers. Formative assessment motivates the students for deep learning.^1^ Frequent summative assessments may render formative assessment insignificant due to less preparation time. Teachers as a facilitator may play an important role in this process of learning and assessment as they help their students to understand their strengths and weaknesses through regular constructive feedbacks.

Assessment at Islamic International Medical College took place after each module. The summative assessment was taken frequently after each module regardless of the length of module and a formative assessment was often planned once in each module for each subject. The students and staff had to bear undue exam pressure most of the time. There was little space available for formative assessment resulting in less chance to improve. Keeping in view the situation, recently a combined modular assessment has been designed: a summative assessment after every three modules with a mid module formative assessment is designed with an aim to augment the learning attitude in students through constructive feed back after each formative assessment. Therefore, students need to appear in two summative block assessments along with one end of year summative assessment. Furthermore, log books are introduced to record the performance in formative and summative assessment.

The present study was designed to determine the perception of students about transition from frequent modular to combined block assessments and its possible positive effects on deep learning.

## METHODS

This was a retrospective descriptive study. Separate questionnaire comprising of 8 questions with likert scale (1-5) was designed to compare the efficacy of CBA with frequent modular exam. The questions were asked in three different grouped for each modality.

Group 1- Learning required in combined block assessment and frequent modular exam.

Group 2- Time for personal development (extracurricular activities).

Group 3- Assessment tools.

Crohnbach ∞ was used to calculate the reliability of questionnaire (0.98). Target students were 3^rd^ year students as these students had given frequent modular exams during 1^st^ year and CBA during 2^nd^ year MBBS. Twenty minutes were given to fill the questionnaire. The received data of questionnaire (Likert scale 1-5) was merged into three categories (agree, do not know, disagree) on Microsoft excel. Then the final data analyzed by using SPPS 21. Chisquare test was done to compare the each corresponding question of two questionnaires. Final results of 1^st^ and 2^nd^ year were obtained from exam department and analyzed for any improvement in result.

**Fig.1:**
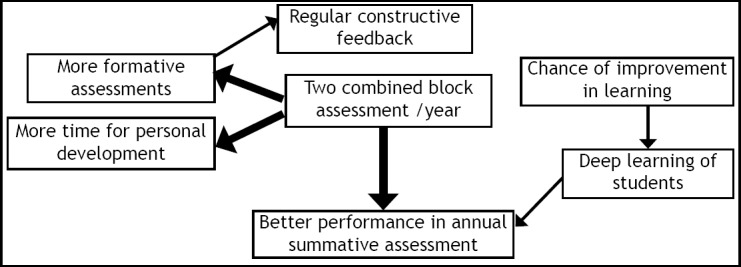
Effect on learning and personal development of the students.

## RESULTS

Transition from frequent modular to CBA has shown positive effects on learning and personal development of the students. The students agreed (60%) that they need to study regularly in frequent modular exams. Multiple revisions were compulsory for better performance and in CBA (55%) students had more chance to study as more time was given in CBA. Therefore, 51% students agreed that indepth studies were mandatory in CBA (Table-I). Evaluation of the type of learning methods display a fact that CBA promotes more in depth studies as compared to modular model and multiple revisions were required to appear in CBA’s. Learning and personal grooming may go side by side due to more time space between two summative assessments Forty two percent students, in comparison with 33%, agreed that CBA helped in their personal development. Thus the environment during the CBA method of assessment was more conducive for the studies as well as for the extracurricular activities.

**Table-I:** Comparison of the percentage of agreement by the students on learning and personal development in combined block assessment and modular exam.

Questions asked		Agree	Disagree	Not sure	P value
*Learning*					
Timekeeping in study was required	CBA	55	34	11	0.00
	In modular exam	60	35	5	
Promotes indepth study	CBA	51	37	12	0.00
	Modular exam	31	63	6	
Multiple revisions are mandatory to appear	in CBA	55	41	4	0.00
	Modular exam	26	61	13	
*Personal development*					
Assists in personal development of the students	CBA	42	45	13	0.00
	Modular exam	33	47	20	

In Combined block assessment with frequent formative assessment MCQ and SEQ along with OSCE were used as an exam strategy: 42% students agreed that this strategy was more helpful in concept building and was statistically significant while only 28% students agreed that MCQS alone were sufficient for their assessment of learning. Therefore, 47% students agreed that assessment tools in CBA were useful for deep learning. In addition to assessment tool, time given for exam preparation was also found helpful as 47% students were in the favor of CBA in comparison with 29% students who agreed that time given was enough in frequent modular exam.

The comparison of final summative assessment results showed improvement in the mean (Table-III) of total marks obtained in combined block assessment. There was decrease in the number of students who could not pull off required passing marks.

**Table-II:** Comparison of the percentage of agreement by the students on efficacy of assessment tools used in combined block assessment and modular assessment.

	Questions asked		Agree	Disagree	Not sure	P value
Efficacy of assessment tool	SEQ* and MCQ** strategy lead to concept building***	In CBA	42	32	27	0.00
	MCQ with OSCE were enough concept building	In modular exam	28	51	21	
	promoted deep learning	CBA	47	43	11	0.00
		modular exam	29	57	15	
	preparatory leaves were sufficient	CBA	47	36	10	0.00
		in modular exam	35	48	16	

* MCQs. Multiple choice questions.

** SEQs. Structured essay questions.

*** Combination of frequent formative and infrequent summative assessment.

**Table-III:** Comparison of the end of year results of same class in the modular and combined block assessment method.

		Mean	SD	Median	Mode	Minimum	Maximum	Fail	Pass	Total
Final results of first year MBBS in modular exams	Paper 1	56	7	57	54	38	78	14	87	101*
	Paper 2	58	9	59	60	24	78	13	88	101*
Final results 2^nd^ year MBBS in CBA	Paper 1	69	8	68	66	25	85	3	92	95**
	Paper 2	62	8	62	61	34	82	3	92	95**

* Number of students in first year 101.

** Number of students was decreased (95) in second year.

## DISCUSSION

A well planned assessment strategy has many positive outcomes ranging from student learning to judgment of competencies. In modular system, Competences are assessed in an integrated logical manner by using compound assessment methods and regular frequent constructive feedback.^8^

Summative assessment is outcome focused as the students have the tendency to study which is going to be tested while with formative assessment if given proper feedback, it focuses on the process of learning reinforcing a student’s inner motivation to study.^8^ Premeditated and correctly implemented assessment strategy has a strong steering effect on learning of students as well as on curriculum. Therefore, regular formative assessments with constructive feedback may improve learning and enable the students to perform better in summative assessment. Reliability, validity, impact on future learning and practice, acceptability to learners and faculty, and costs are five factors which may determine the efficacy of an assessment method.^9^ Certain limitation in resources like time required to develop and take test followed by grading the papers are important. Cost associated with exams and faculty training is equally important. In modular teaching system, there is an established link among frequency, format, content and timing of formative and summative assessment.^8^ Thus, keeping in view these factors, every medical school may analyze and implement a modified assessment strategy based on fewer summative and frequent formative assessments.

Mode of assessment determines the student’s approach towards learning and may affect their pattern of study. There is a strong relationship between learning strategy and academic tasks given to students. Excessive workloads and inappropriate mode of assessment may lead to superficial learning and may also provoke negative attitude in students.^10^ In this way student will not develop insight into context.

Deep learning is associated with regular formative assessment and constructive feedback: it is a great tool for professional development.^11^ It enables a student to have better concepts and to correlate the basic and clinical sciences knowledge. (Table I and II). Regular and repeated formative assessment provide yardstick for proper orientation of the learner having relatively immature knowledge. The students will get motivation to learn more by setting higher standards for themselves.^3^ In the modular exam modal, formative assessments were found to be insignificant as teachers did not have enough time for constructive feedback and suggested remedial were not taken well by students due to pressure of frequent summative exams. The students agreed transition from regular frequent modular exam to combined block assessment with associated regular formative assessment helped the student in improving their grades (Table-III).

When frequent summative assessments are held during an academic year the students face more burdens and may also result in rote learning of the subject by the students instead of developing a deeper understanding. Moreover, frequent exams also might put undue pressure on staff. In combined modular assessment, multiple revisions ensured the conceptual deep learning with personal grooming of each student. Therefore, the scheduling of exams is very important: rather than conducting many summative assessments over short period, infrequent assessments should be contemplated to produce breaks between exams.

Assessment tools also affect the learning process, although multiple choice questions of clinical context were used both in CBA and modular exams as they are thought to be standard method of exam as they trigger the cognitive processes in students.^12^ but students akin to the addition of structured essay questions in formative assessment which stipulate the contextual learning by students. Furthermore, log books helped to document and reflect the performance of students.^13^ Regular formative assessments and portfolio helps the students to study regularly and improve their learning while preparing for summative assessment.

## CONCLUSION

Timing of assessment is a very important issue. Transition from frequent modular to combined modular assessment may relieve the students from undue pressure. Regular formative assessment with constructive feedback during the modules can help the students for complex structured learning.
